# Family Caregivers of Persons With Mild Dementia Share Their Spiritual Struggles

**DOI:** 10.1093/geroni/igab046.1952

**Published:** 2021-12-17

**Authors:** Jocelyn McGee, Davie Morgan, Dennis Myers

**Affiliations:** 1 Baylor University, United States; 2 Baylor University, Waco, Texas, United States

## Abstract

The lives of family caregivers of persons with Alzheimer’s disease and related dementias (ADRD) may change dramatically with disease progression in their loved one. Many rely on spirituality as a resource for coping. There is evidence that persons experiencing transition/losses, as a consequence of disease/illness, can experience spiritual struggles or a crises in meaning. However, there is limited research related to spiritual struggles among family caregivers of persons with ADRD, particularly in the beginning stages of the disease process. In this study, three domains of spiritual struggle were identified after analyzing 27 caregiver interviews using the constant comparative method: 1) changes in relationship with their higher power (e.g., feelings of anger towards, feeling punished by, feeling disconnected from, and questioning); 2) changes in spiritual practices (e.g., decreased participation as a consequence of feeling unsupported, judged, or misunderstood by spiritual communities); and 3) dissonance between previously held core beliefs and current life circumstances (e.g., feelings of shame, doubt, and guilt as well as cessation of self-care activities due to the belief that they must sacrifice everything for their loved one). Notably, 74% experienced spiritual struggle in one domain; 33% in two domains, and 11% in three domains. The majority of participants had come to resolution of these spiritual struggles by the time they were interviewed. However, 40.7% were experiencing ongoing spiritual struggles, at the time of interview, suggesting the importance of identifying and addressing spiritual struggles in this population over time in order to enhance coping and adaptation.

